# deepBlastoid: a deep learning model for automated and efficient evaluation of human blastoids

**DOI:** 10.1093/lifemedi/lnaf026

**Published:** 2025-07-11

**Authors:** Zejun Fan, Zhenyu Li, Yiqing Jin, Arun Pandian Chandrasekaran, Ismail M Shakir, Yingzi Zhang, Aisha Siddique, Mengge Wang, Xuan Zhou, Yeteng Tian, Peter Wonka, Mo Li

**Affiliations:** Bioengineering Program, Biological and Environmental Science and Engineering Division (BESE), King Abdullah University of Science and Technology (KAUST), Thuwal 23955, Saudi Arabia; Bioscience Program, Biological and Environmental Science and Engineering Division (BESE), King Abdullah University of Science and Technology (KAUST), Thuwal 23955, Saudi Arabia; Computer Science Program, Computer, Electrical and Mathematical Science and Engineering Division (CEMSE), King Abdullah University of Science and Technology (KAUST), Thuwal 23955, Saudi Arabia; Bioscience Program, Biological and Environmental Science and Engineering Division (BESE), King Abdullah University of Science and Technology (KAUST), Thuwal 23955, Saudi Arabia; Bioscience Program, Biological and Environmental Science and Engineering Division (BESE), King Abdullah University of Science and Technology (KAUST), Thuwal 23955, Saudi Arabia; Bioscience Program, Biological and Environmental Science and Engineering Division (BESE), King Abdullah University of Science and Technology (KAUST), Thuwal 23955, Saudi Arabia; Bioscience Program, Biological and Environmental Science and Engineering Division (BESE), King Abdullah University of Science and Technology (KAUST), Thuwal 23955, Saudi Arabia; Bioscience Program, Biological and Environmental Science and Engineering Division (BESE), King Abdullah University of Science and Technology (KAUST), Thuwal 23955, Saudi Arabia; Bioscience Program, Biological and Environmental Science and Engineering Division (BESE), King Abdullah University of Science and Technology (KAUST), Thuwal 23955, Saudi Arabia; Bioscience Program, Biological and Environmental Science and Engineering Division (BESE), King Abdullah University of Science and Technology (KAUST), Thuwal 23955, Saudi Arabia; Bioscience Program, Biological and Environmental Science and Engineering Division (BESE), King Abdullah University of Science and Technology (KAUST), Thuwal 23955, Saudi Arabia; Computer Science Program, Computer, Electrical and Mathematical Science and Engineering Division (CEMSE), King Abdullah University of Science and Technology (KAUST), Thuwal 23955, Saudi Arabia; Bioengineering Program, Biological and Environmental Science and Engineering Division (BESE), King Abdullah University of Science and Technology (KAUST), Thuwal 23955, Saudi Arabia; Bioscience Program, Biological and Environmental Science and Engineering Division (BESE), King Abdullah University of Science and Technology (KAUST), Thuwal 23955, Saudi Arabia; KAUST Center of Excellence for Smart Health (KCSH), Thuwal 23955, Saudi Arabia

**Keywords:** embryonic development, deep learning, blastoid, pluripotent stem cells, image-based classification

## Abstract

Recent advances in human blastoids have opened new avenues for modeling early human development and implantation. Human blastoids can be generated in large numbers, making them well-suited for high-throughput screening. However, automated methods for evaluating and characterizing blastoid morphology are lacking. We developed a deep-learning model—deepBlastoid—for automated classification of live human blastoids using only brightfield images. The model processes 273.6 images per second with an average accuracy of 87%, which is further improved to 97% by integrating a Confidence Rate metric. deepBlastoid outperformed human experts in throughput while matching accuracy in blastoid classification. We demonstrated the utility of the model in two use cases: (i) systematic assessment of the effect of lysophosphatidic acid (LPA) on blastoid formation and (ii) evaluating the impact of dimethyl sulfoxide (DMSO) on blastoid formation. The evaluation results of deepBlastoid using over 10,000 images were consistent with the known drug effects and showed subtle but significant effects that might have been overlooked in manual assessments. The publicly available deepBlastoid model enables researchers to train customized models based on their imaging and protocols, providing an efficient, automated tool for blastoid classification with broad applications in research, drug screening, and *in-vitro*-fertilization applications.

## Introduction

Mammalian development begins with a fertilized egg that has the potential to form all embryonic [1–3] and extra-embryonic lineages [4–6]. As development progresses, cells within the embryo gradually lose their potency and begin to differentiate into specialized cell types [7]. In the human embryo, the earliest cell fate decisions are made by the time of blastocyst formation [8], just prior to implantation.

**Figure 1.**
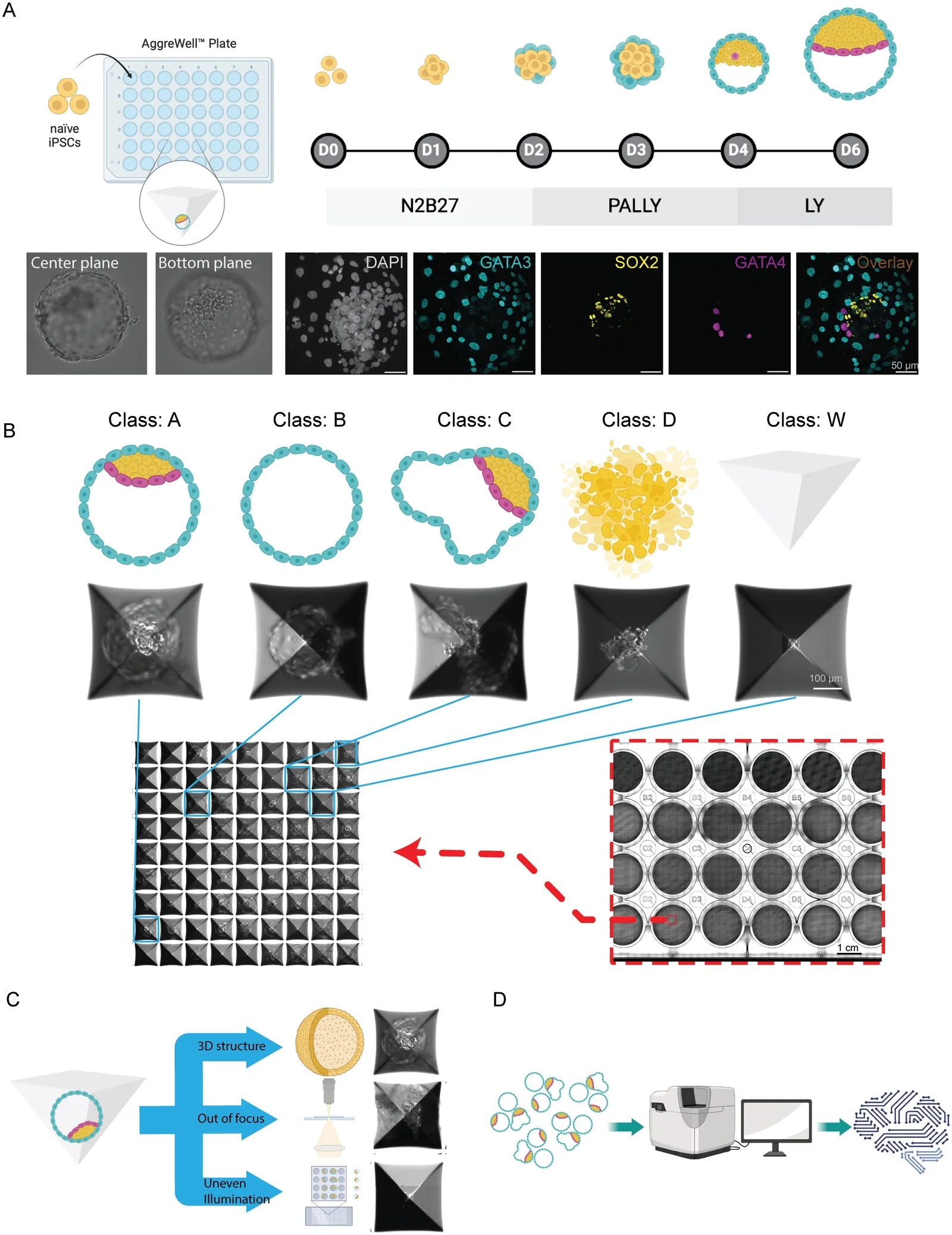
Generation of a blastoids image dataset for our classification model. (A) Protocol of blastoid culture and immuno-fluorescence microscopy images of blastoids. The culture medium, N2B27, PALLY and LY is followed by an established protocol, Ref. [15]. D0 is the date of cell seeding. Brightfield images of blastoid in different planes. The blastoids were fluorescently labeled with DAPI (gray), GATA3 antibody (magenta), SOX2 antibody (yellow) and GATA4 antibody (cyan). Scale bar, 50 μm. (B) Five typical classes of blastoids generated in AggreWell. Top: schematic of different classes of blastoids. Middle: 2D brightfield images of blastoids in microwell. Scale bar, 100 μm. Bottom: generation of blastoids in AggreWell plate. Each plate has 24 wells. Each well has about 1200 individual microwells for blastoids formation. Scale bar, 1 cm. (C) Schematic of three common interferences hindering traditional image analysis in blastoids classification. (D) Schematic representation of the assay workflow used to generate the original blastoid images. The cells are seeded in the AggreWell Plate and harvested at Day 6. Images are acquired by CellDiscoverer7 microscopy, followed by AI model training.

The blastocyst, which emerges approximately three and a half days post-fertilization (E3.5) in mice and around five days in humans, is defined by a fluid-filled cavity and consists of three distinct cell populations [9]: the outer extra-embryonic trophectoderm (TE), and the inner epiblast (EPI) and primitive endoderm (PrE), which are positioned adjacent to the cavity. Due to ethical considerations of experimenting with human embryos, alternative models have been developed to recapitulate many aspects of the human peri-implantation development [10, 11]. Recent studies [12–21] have demonstrated that stem cells can self-organize *in vitro* to generate blastocyst-like structures, termed blastoids (Fig. 1A). By closely mimicking early human embryonic stages, blastoids offer an ethical and cost-effective alternative for understanding the mechanisms underlying embryogenesis, investigating pregnancy-related diseases such as abnormal development and pregnancy failure, and testing drug safety and efficacy in a controlled environment [22, 23].

Over the past year, various methods [12, 13, 15, 16, 24] for generating human blastocyst models have been proposed, with most being implemented in the AggreWell format [15, 25]. However, automatic characterization of blastoids remains largely underdeveloped. Key parameters, such as cavitation efficiency, are still manually evaluated by researchers, and large amounts of experimental data remain underexplored, leading to missed opportunities for deeper insights [26, 27]. In a study by Rivron group [15], at most, a few hundred blastoids were manually analyzed per experiment, despite an AggreWell plate containing about 30,000 microwells for blastoids, a trend also observed in studies by others [12, 18]. Applications such as genetic and drug screening demand high-throughput, rapid evaluations, yet the lack of automated characterization tools often leads to valuable data being underutilized [28].

Deep learning has revolutionized image evaluation, leading to major advancements in various fields, including biomedicine, by enabling accurate disease diagnosis [29], drug discovery [30], and personalized treatment strategies [31] through models, such as ResNet, EfficientNet, DenseNet, and ViT. While ResNet-based models such as EmbryoNet [32] have identified molecular defects in zebrafish embryos, and StembryoNet [33] has detected abnormalities in mouse stem cell-derived embryo models using fluorescent images, there is still no automated deep-learning model specifically designed for human stem cell-derived embryo model evaluation. Existing deep learning approach [33] primarily depends on genetically modified cells, limiting the universal applicability for mechanism studies and translational applications.

Here, to address these challenges, we developed a deep-learning classifier called deepBlastoid, based on a fine-tuned ResNet-18 [34–36] model, for efficient identification and classification of blastoids. deepBlastoid, using only brightfield images, is capable of automatically classifying blastoids into five distinct morphological categories. The trained classifier processes an average of 273.6 images per second with an accuracy of 87%. As a standardized platform, researchers can select an appropriate Confidence Rate (CR), which flags low-confidence classifications for human expert review, thereby increasing overall accuracy to 97%, albeit with a slight reduction in processing speed. We demonstrate two real-world applications of this platform using wet lab experiments: (i) analyzing the effect of lysophosphatidic acid (LPA) dosage gradients on blastoid formation across different classes, and (ii) assessing the impact of dimethyl sulfoxide (DMSO), a commonly used pharmaceutical solvent [37, 38], on blastoids for drug screening. deepBlastoid confirmed the known effect of LPA dosage on blastoid formation and showed subtle but significant effects induced by low-dose LPA and DMSO that might have been overlooked in manual assessments. The model facilitated additional quality assurance assessment of blastoid experiments, thereby strengthening the reproducibility of the experiment.

In summary, deepBlastoid provides a detailed and automated classification platform for evaluating blastoids, uncovering new insights into developmental mechanisms [39, 40]. For drug screening [41–43], the platform offers an efficient, automated, and easy-to-use solution, enabling large-scale screening of human blastoid models.

## Results

### Establishment of a blastoid dataset

Among all blastoid generation methods, we chose to generate human blastoid from naïve iPSCs following the PALLY blastoid protocol [15] (Fig. 1A). Blastoid formation using the PALLY protocol entails stepwise medium inductions over 6 days following the seeding of naïve iPSCs in an AggreWell plate (Fig. 1A). During the first 2 days, naïve iPSCs are cultured in the N2B27 medium, allowing initial aggregation and differentiation. On days 3–4, the medium is switched to PALLY, which drives lineage specification into epiblast (EPI) and trophectoderm (TE), facilitating the cavity formation and spatial organization. In the final two days (days 5–6), cells are cultured in LY medium, promoting further differentiation of the EPI, TE and primitive endoderm (PrE) lineages, resulting in a blastoid that closely resembles a preimplantation blastocyst. Blastoids share several similarities with natural human embryos, including lineage specification, gene expression patterns, and implantation potential *in vitro*. Well-formed blastoids exhibit a characteristic fluid-filled cavity and three cell types—SOX2^+^ EPI, GATA4^+^ PrE, and GATA3^+^ TE—which would develop into the embryo proper, yolk sac, and placenta *in vivo*, respectively (Fig. 1A).

In our previous studies [11, 24], blastoids have been extensively characterized using single-cell RNA sequencing (scRNA-seq), karyotyping, functional assays, and pluripotency assessments. The results demonstrated that the blastoids exhibit post-implantation potential (Fig. S1A). Upon attachment of blastoid, TE cells extend and proliferate outward, closely mimicking early post-implantation embryonic structures.

Each well of a 24-well AggreWell plate contains 1200 microwells, resulting in nearly 30,000 blastoids per plate that require evaluation (Fig. 1B). These blastoids could be categorized into five classes based on expertise of human experts in assessing their morphological features in previous experiments (Fig. 1B). Class A represents a blastoid with a well-formed cavity and an inner cell mass (ICM) enveloped by a complete circular TE layer. Class B represents a blastoid with a cavity but no ICM. Class C represents a blastoid with an ICM but an irregular or broken TE layer. Class D represents a structure that does not contain a cavity, appearing as cellular debris. Class W represents an empty microwell. Automated image-based blastoid classification presents inherent challenges, such as the compression of 3D structures into 2D images, variations in focal plane, and uneven illumination (Fig. 1C). These factors increase analytical complexity and must be carefully considered when designing a deep learning platform.

Brightfield images of each blastoid in microwells were captured using an automated live-cell imaging microscope to create the blastoid image dataset (Fig. 1D). Based on morphological features, images from three independent experiments were classified and labeled into five defined classes by three human experts in three independent experiments (Fig. S1B). Inter-rater variability was assessed across 2407 labeled images, revealing strong agreement and high reliability among the raters. The 2407 labeled images and 14,726 unlabeled images, collected under various experimental conditions, constitute the first curated image dataset of human blastoids and are provided as an open resource (Zenodo; see Methods). It may serve as a valuable asset for future self-supervised learning approaches.

### Training of the deepBlastoid model

**Figure 2.**
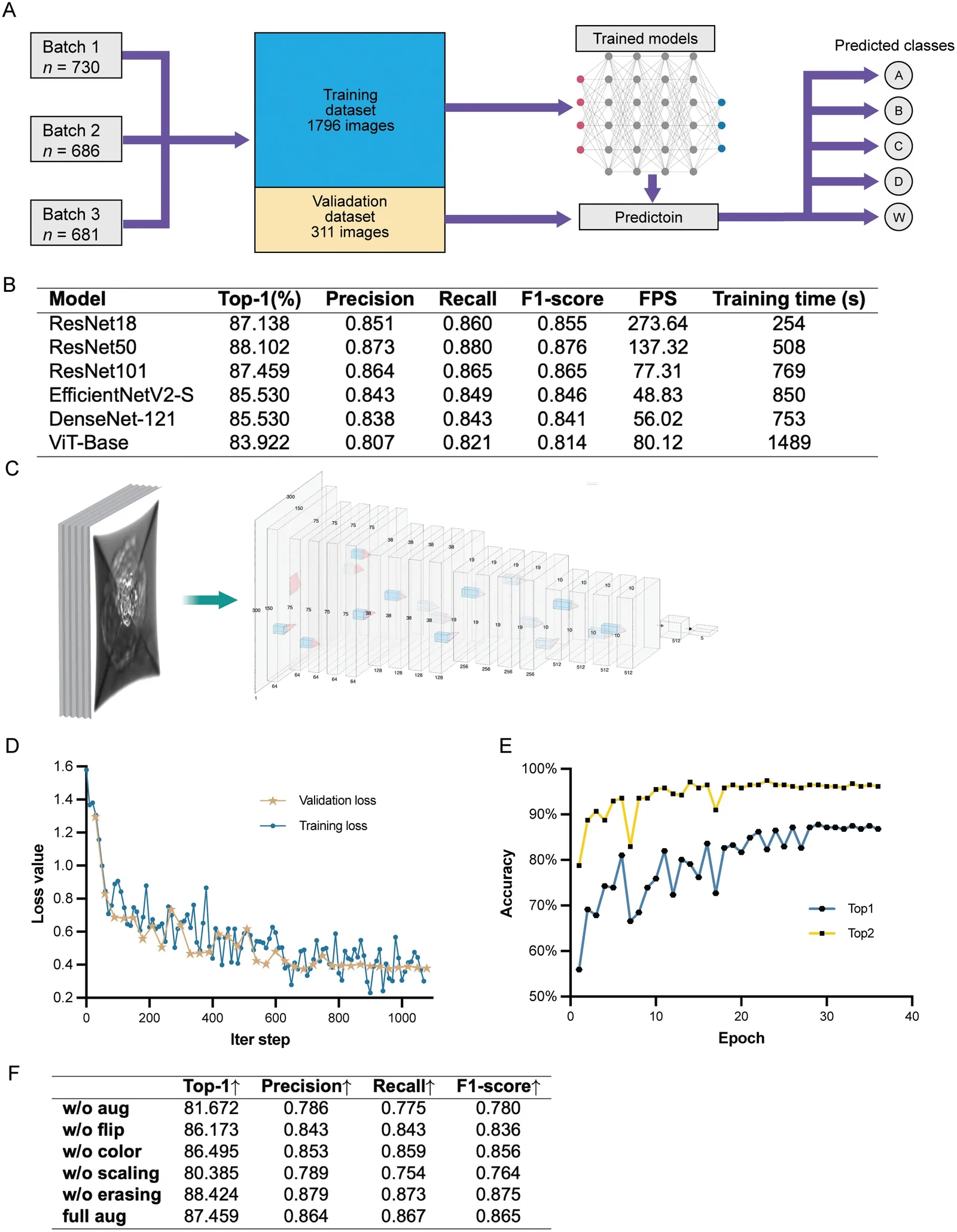
Training of blastoids classification model. (A) Workflow of the model. All images form three batches are evenly divided into training dataset (1796 images) and validation dataset (311 images). Using the training dataset, the model is cross-trained and followed by validation. (B) Table of performance of different models. All models are tested using the same hardware condition listed in Methods. (C) Architecture of deep learning ResNet18. The architecture is composed of seventeen convolutional layers, one max pooling layer, one average pooling layer and one fully connected layer. (D) Representative loss curve versus epoch in training. Validation loss, yellow curve. Training loss, blue curve. Iteration step (Iter step) refers to a single update of the model’s parameters during training, where a batch of data is processed through forward and backward propagation. An epoch represents one complete pass through the entire dataset. In this study, 1 epoch consists of 29 iter steps, meaning the model updates its parameters 29 times per epoch. (E) Top1/Top2 accuracy curves for the validation dataset versus epoch in training. Top-1 accuracy represents cases where the true class is the first prediction, while Top-2 accuracy indicates cases where the true class is within the first two predictions. The accuracy curves are stable after epoch 36. (F) Table of ablation of augmentation in ResNet18 on the validation set. The w/o is for without.

After the collection of the blastoid imaging data (Fig. 1D), the overall data workflow is outlined in Fig. 2A. Among the 2407 labeled images, 300 were used as the test set. The remaining 2107 images were randomly divided into a training set (1796 images) and a validation set (311 images).

Considering the balance between computational efficiency and effectiveness (Fig. 2B), we choose the ResNet architecture as the default classification model in this work. We evaluated several deep learning models—including ResNet variants (18, 50, and 101 layers), EfficientNetV2-S, DenseNet-121, and ViT-Base (based on B32 transformer)—and compared their performance on our dataset. As shown in Fig. 2B, the ResNet architectures offered the best balance between accuracy and inference time. Specifically, although deeper networks like ResNet-50 and ResNet-101 may capture more complex features, they did not significantly improve performance and required greater computational resources, resulting in longer training and inference times (Fig. 2B). Consequently, the choice of architecture should be guided by the complexity of the dataset and the available computational resources. In our study, ResNet-18 emerged as the most effective option.

Given an input image, the ResNet-18 model extracts feature maps through successive convolutional layers with shortcut connections (i.e. residuals) and finally projects the last feature to the classification probability via an average pooling, a fully connected layer and a softmax function (Figs. 2C and S1C, Table S1). These residual connections address the vanishing gradient problem [44], enabling the training of such deeper networks by allowing gradients to flow more effectively through the network during backpropagation [45, 46].

We employed the cross-entropy loss [47, 48] (formula in Methods) and AdamW optimizer to train our model for 36 epochs with a learning rate of 3 × 10^−4^ and a weight decay of 1 × 10^−3^. We clipped the gradients to stabilize the training and scaled the learning rate of the last fully connected layer by 10 to achieve faster convergence. The validation loss value decreased with some volatility during training and eventually plateaued after 696 iter steps (24 epochs), indicating the point at which the model was well-trained (Fig. 2D). Similarly, on the validation set, both Top-1 accuracy (cases where the true class is the first prediction) and Top-2 accuracy (cases where the true class is among the top two predictions) followed a similar trend, reaching a plateau after 24 epochs (Fig. 2E). We thus selected the model trained with 36 epochs to ensure convergent.

There are various data augmentation strategies commonly used in image classification tasks during the training stage, including horizontal and vertical flipping [49], color jitter [50], random scaling (resizing the input image), and random erasing [51]. To diversify the training data and further improve the model performance and generalization, we evaluated these data augmentation strategies to determine their impact on model performance. Most augmentation methods improved the F1-Score compared to the baseline model without augmentation. Notably, random scaling was the most effective, whereas random erasing negatively affected the model’s performance (Fig. 2F). We also tested if 5-fold cross-validation might improve the performance of ResNet-18 (Fig. S1D). However, we observed a slight drop in validation performance with 5-fold cross-validation—likely because each fold contained fewer training samples (4/5 dataset, 1687 images) compared to our default split (1796 images). Consequently, we opted for our default train-validation split in this study. We also experimented with a round-robin approach where we trained on data from two batches and used the remaining batch as the validation set. However, no performance gain was observed using this approach (Fig. S1E).

### Performance of the deepBlastoid model

**Figure 3.**
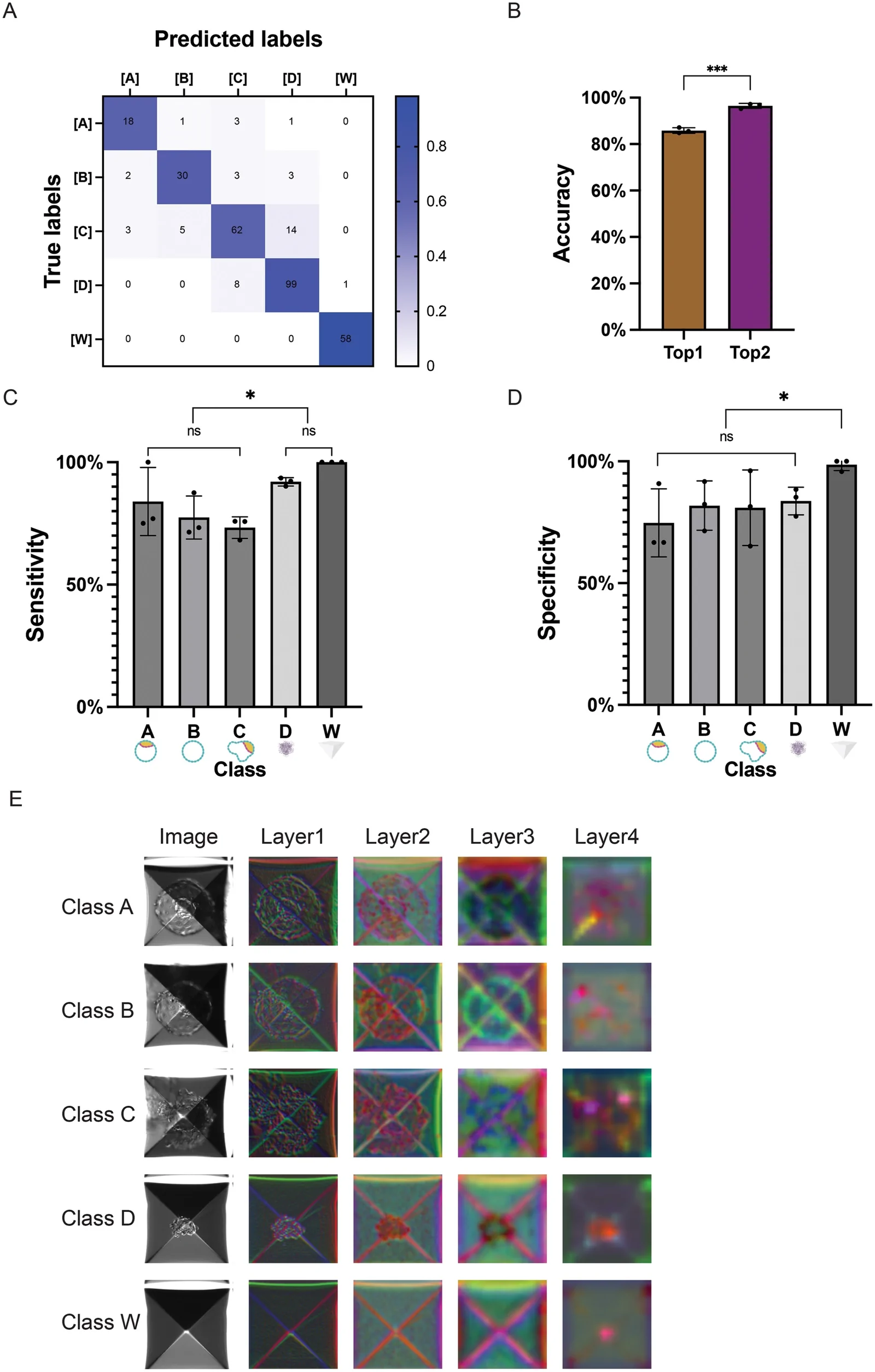
Performance of the blastoids classification model. (A) Confusion matrix on the test set from three batches. Each cell shows the number of samples classified into each category. The color in cells illustrates relative distribution intensity. (B) Top-1/Top-2 accuracy of model. Top-1 accuracy represents cases where the true class is the first prediction, while Top-2 accuracy indicates cases where the true class is within the first two predictions. Specificity (C) and sensitivity (D) of model for five classes. Specificity is for evaluation of false positives and sensitivity is for evaluation of false negatives. Each point is from an individual batch dataset. (E) Feature visualization of each class in different layers. *P < 0.05, **P < 0.01, ***P < 0.001, ****P < 0.0001.

We next evaluated the performance of the trained ResNet-18 model, hereafter referred to as deepBlastoid. The confusion matrix for the validation dataset (Fig. 3A) provided a detailed evaluation of the deep learning classifier’s performance by displaying the number of correct and incorrect predictions for each class. The intensity of the blue shades in the confusion matrix reflects the relative distribution of predictions, with the highest values along the diagonal—indicating that most images were correctly classified. However, some misclassifications occurred, particularly between classes C and D, likely due to their shared biomorphological features.

Overall, the classifier achieved a Top-1 accuracy of approximately 87% and a Top-2 accuracy of 97%, with highly reproducible performance across the three experimental batches (Fig. 3B). In addition, intuitive metrics such as specificity and sensitivity (Fig. 3C and 3D) further supported the model’s robust performance. Sensitivity and specificity values were consistent across batches. The model demonstrated consistent sensitivity in classifying Classes A, B, and C, while its sensitivity was significantly higher for Classes D and W (Fig. 3C), consistent with the confusion matrix (Fig. 3A). For specificity, the model performed similarly when classifying Classes A, B, C, and D, while showing significantly higher specificity for Class W (Fig. 3D). This superior classification performance for Class W may be attributed to its distinct and easily recognizable features. These results revealed a consistent accuracy across batches, supporting a robust model architecture and an optimal number of training epochs. Together, these comprehensive metrics provide a thorough evaluation of the model’s performance.

To understand how the deepBlastoid model perceives and classifies images, we performed a feature importance analysis by visualizing the intermediate feature maps of the model (Fig. 3E). Our findings revealed that the shallow layers (e.g. layer 1 and 2) primarily captured high-frequency details, whereas the deeper layers (e.g. layer 3 and 4) focused on broader, low-frequency structures. Notably, in the deepest layers (e.g. layer 4), the model appeared to pay special attention to the center of the grid. Additionally, certain types of noise, such as uneven illumination, were filtered out in layer 1, whereas structural artifacts like microwell diagonals and cropping mismatches were mitigated in layer 3 and 4. deepBlastoid also exhibited varying regions of attention depending on the blastoid class, focusing on irregular outlines in Class C, the boundary between ICM and TE in Class A, and central debris in Class D. This special attention also corresponds to the hallmark process of blastoid formation, cavitation, which generally is observed in the center of the image. These visualizations provide insights into the salient features that inform the model’s decisions, enhancing its interpretability.

### Throughput and accuracy with different confidence rate

**Figure 4.**
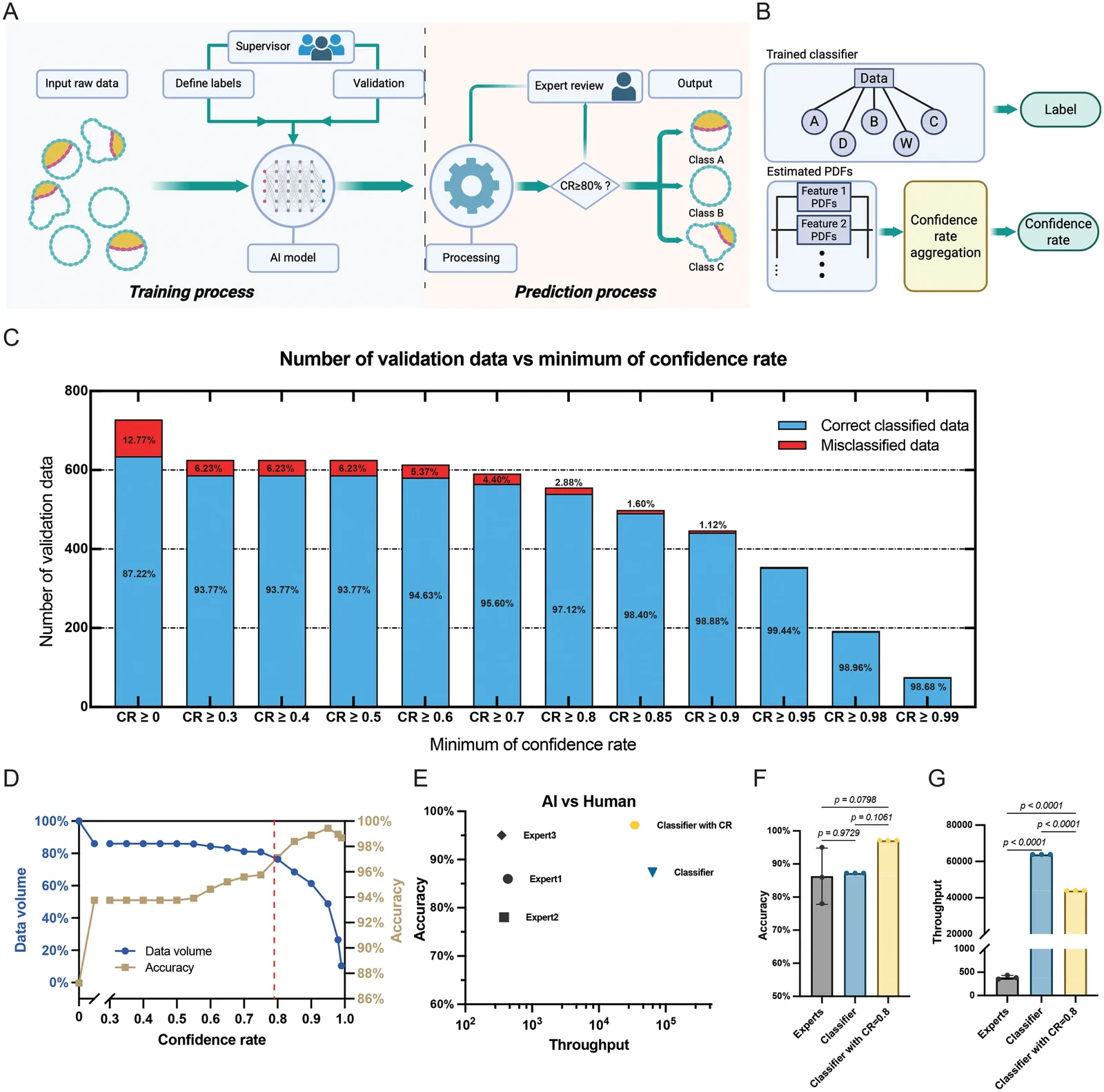
Classifier of blastoids images with confidence assessment. (A) Data flow of classifier with CR and human expert review. In this case, the supervisor is three biological researchers, who labeled images based on their professional expertise. (B) Schematic of proposed classifier with CR. The trained hierarchical classifier selectively utilizes features for labeling purposes. A misclassification probability is assigned to each feature using the estimated Probability Density Functions (PDFs). These probabilities are aggregated to a confidence rate for the concluded label. (C) The performance of the proposed classifier with CR on an independent dataset. (D) Relationship between minimum CR and data volume & accuracy. Beige curve is for accuracy. Blue curve is for data volume. Red dot line is for the intersection where CR = 0.8. (E) Comparison of performance between Deep learning model and human experts in throughput and accuracy. The *x*-axis is for the number of images processed by AI or human in 20 min. In the Classifier with CR, 0.8 is chosen as an acceptable confidence rate. (F) Quantitative analysis of accuracy performance of human experts and Deep learning model. The dots represent the performance of three human experts and the model, which was run three times on the same dataset. The repeated runs produce similar values due to the deterministic nature of the model. (G) Quantitative analysis of throughput of human experts and Deep learning model.

While deepBlastoid achieved satisfactory accuracy, we sought to further reduce misclassification and enhance its performance in real-world biological applications. To accomplish this, a CR metric was introduced to filter out images likely to be misclassified, directing them to human experts for review (Fig. 4A). For each input image, the deepBlastoid model outputs probabilities for all potential classes, which are then integrated to calculate the CR. The model generates both the preliminary prediction and the CR simultaneously (Fig. 4B).

As the CR threshold increases, the volume of misclassified data decreases, boosting accuracy from 87% to 99% (Fig. 4C). However, this improvement significantly reduces the size of the automatically processed dataset, thereby shifting more workload to human reviewers and diminishing practical efficiency. The CR threshold can be chosen according to the demand for accuracy and throughput. As depicted by the yellow accuracy curve and the blue data volume curve in Fig. 4D, a CR of 0.8 may serve as an optimal minimum threshold to identify unreliable samples. At this point, the model’s accuracy rises substantially from 87% to 97%, while avoiding the steep decline in data volume. The implementation of CR is an optional feature that provides users with the flexibility to adjust the model to meet the accuracy and throughput requirements of specific applications.

### Comparison with human experts

To evaluate throughput and accuracy, we compared the deepBlastoid classifier to three human experts using an independent dataset, containing 672 images. deepBlastoid demonstrated high processing speed—with throughput approximately 1000 times greater—and accuracy comparable to that of human experts (Fig. 4E). In an AggreWell experiment with 30,000 blastoids, the model processed the data within just a few minutes. Additionally, when combined with the CR feature, deepBlastoid maintained high accuracy (Fig. 4F) while achieving significantly higher throughput (Fig. 4G).

To demonstrate the real-world utility of the deepBlastoid model in human blastoid research, we next applied it to two use cases addressing genuine research needs.

### Use case I: LPA dosage optimization for blastoid formation

**Figure 5.**
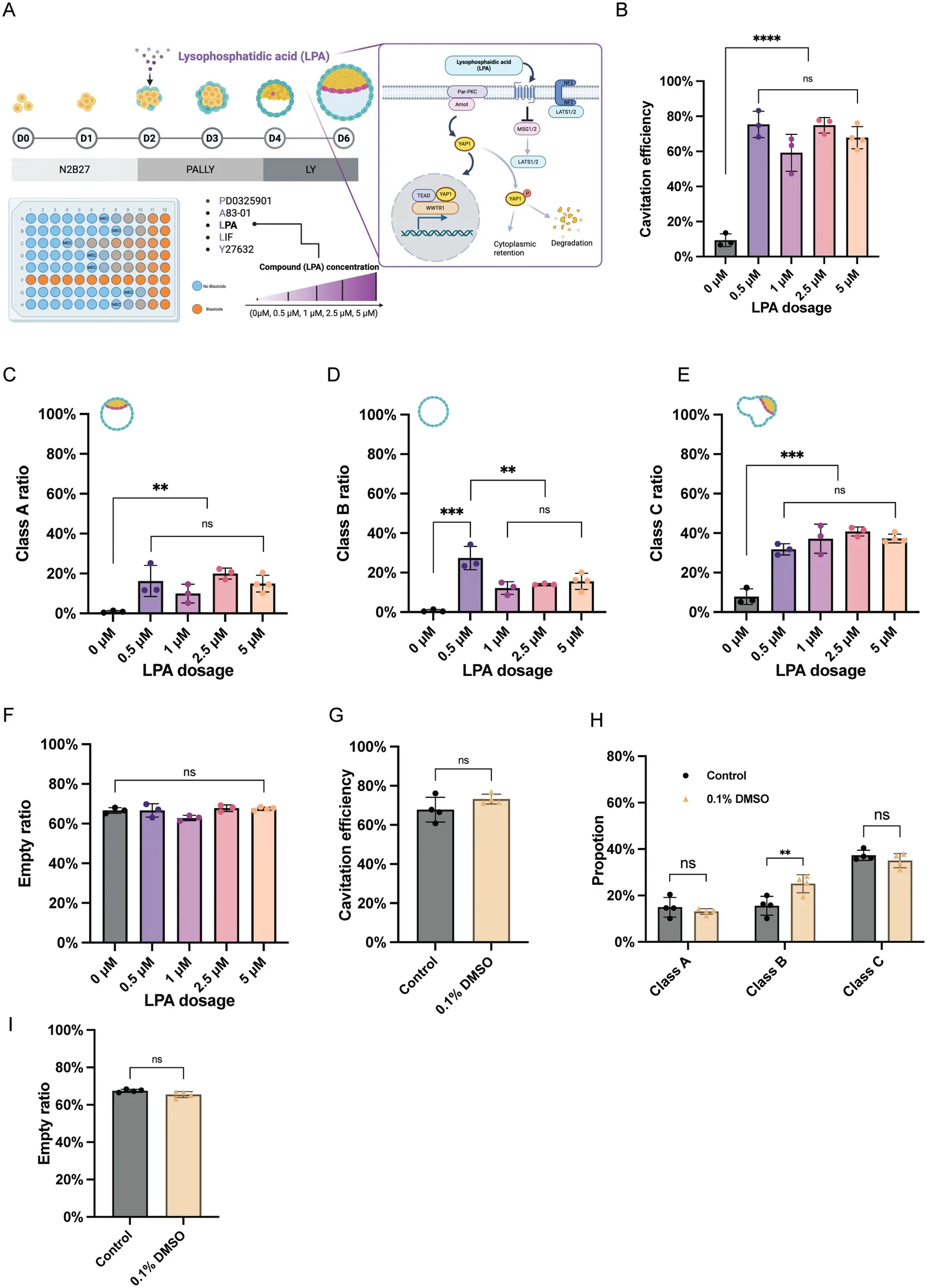
Application of classifier model for blastoids. (A) Schematic of dose-effect experimental design of LPA on blastoids formation, including 0 μM, 0.5 μM, 1 μM, 2.5 μM and 5 μM. All culture conditions are normal, except LPA concentration. Purple box indicates a diagram about LPA and its role in blastoid formation by inhibiting the Hippo pathway. LPA has a significant dose-effect on blastoids formation in the aspect of cavitation efficiency (B), class-A efficiency (C), class-B efficiency (D), class-C efficiency (E). Cavitation efficiency is calculated as the total ratio of Class A, B, and C combined. (F) Empty ratio in the gradient LPA dosage experiment. Empty ratio is the frequency of Class W events. It can serve as a quality control to monitor the experimental process. (G) Cavitation efficiency of blastoid formation in the 0.1% DMSO experiment. (H) Effects of 0.1% DMSO on blastoids formation in different classes. Each point is for an independently replicated experiment. One-way ANOVA and Tukey’s multiple comparisons test. (I) Empty ratio in the 0.1% DMSO experiment. *P < 0.05, **P < 0.01, ***P < 0.001, ****P < 0.0001.

During blastoid formation, various inhibitors and growth factors are employed to induce the differentiation of cell lineages that resemble the TE, EPI, and PrE of blastocysts. The concentrations of these factors require careful titration in protocol development and optimization when using different cell lines [16]. However, systematic optimization is time-consuming and labor-intensive and is often abbreviated in practice, leading to suboptimal conditions. One of such factors, LPA, a key compound in the PALLY medium [25], plays a crucial role in inducing blastoid formation [52, 53]. LPA acts through its receptors to regulate various cellular processes, including the inhibition of the Hippo pathway during TE development [54, 55] (Fig. 5A). We aimed to test whether the deepBlastoid model can reduce manual efforts and provide a more efficient, reproducible evaluation of blastoid formation. To do this, we conducted experiments using varying LPA dosages for blastoid generation (Fig. S2A–E) and applied the deepBlastoid model to analyze the results. A gradient of LPA doses, including 0, 0.5, 1, 2.5, and 5 µM, was tested, and over 10,000 images from three independent batches were processed by the deepBlastoid classifier (Fig. 5A). Cavitation efficiency was calculated as the combined frequency of Class A, B, and C. Consistent with previous studies [15], cavitation efficiency increased with higher LPA dosages, with the minimum effective concentration (MEC) being 0.5 µM. Beyond 0.5 µM, no significant differences were observed in cavitation efficiency (Fig. 5B).

Additionally, deepBlastoid revealed an increased frequency of Class A, B, and C blastoids wherever LPA was supplemented (Fig. 5C–E). Different LPA dosages did not affect the frequency of Class A and C blastoids. Notably, a significant two-fold increase in Class B frequency was observed at the 0.5 µM dosage compared with 1 µM, 2.5 µM, and 5 µM. This suggests that 0.5 µM may represent an optimal concentration for TE cavitation, likely because it provides sufficient signaling to support TE differentiation and lumen formation [54] without inducing overstimulation or cytotoxic effects [53]. Without the automated classification by deepBlastoid, such observations could have been overlooked. Further biological experiments are necessary to investigate the underlying mechanisms behind this finding.

In this application, we employed the “empty ratio,” defined as the frequency of Class W events, as a metric to evaluate cell seeding density, which serves as a quality control measure. During the calculation and calibration process (Fig. S1F), we identified a correlation between the empty ratio and cell seeding density, with the empty ratio decreasing as seeding density increased, and vice versa. In one calibration experiment, the predicted density of 73 cells per microwell was in close agreement with the actual density of 75 cells per microwell, thereby confirming the empty ratio as a reliable indicator of cell seeding density. No significant differences in empty ratio were detected across experimental batches along the LPA gradient (Fig. 5F), underscoring the consistency and reliability of the overall experimental setup and ensuring the robustness of the deepBlastoid results.

### Use case II: the effect of DMSO on blastoid formation.

DMSO (dimethyl sulfoxide), commonly used as a chemical solvent, is often present in low concentrations during clinical therapy and drug screening [37]. However, the impact of DMSO, or like compounds used in clinical or research setting, on blastoid formation is only starting to be studied [24]. We hypothesized that deep learning models such as deepBlastoid can aid the efficient evaluation of potential effect compounds (Fig. S3A and S3B) on blastoid formation. As a proof-of-principle, we next used the deepBlastoid classifier to investigate the effect of low-dose DMSO on blastoid formation. There was no significant difference between the control group and the 0.1% DMSO group in terms of overall cavitation efficiency (Fig. 5G), Class A, and Class C (Fig. 5H). Interestingly, the efficiency of Class B blastoids was higher in the 0.1% DMSO group, suggesting that DMSO might promote TE differentiation. Overall, low-dose DMSO does not affect blastoid cavitation efficiency (Fig. 5G), consistent with a previous study [24]. The similar empty well ratios between the control and 0.1% DMSO groups confirm the reliability of the experiment (Fig. 5I).

## Discussion

**Figure 6.**
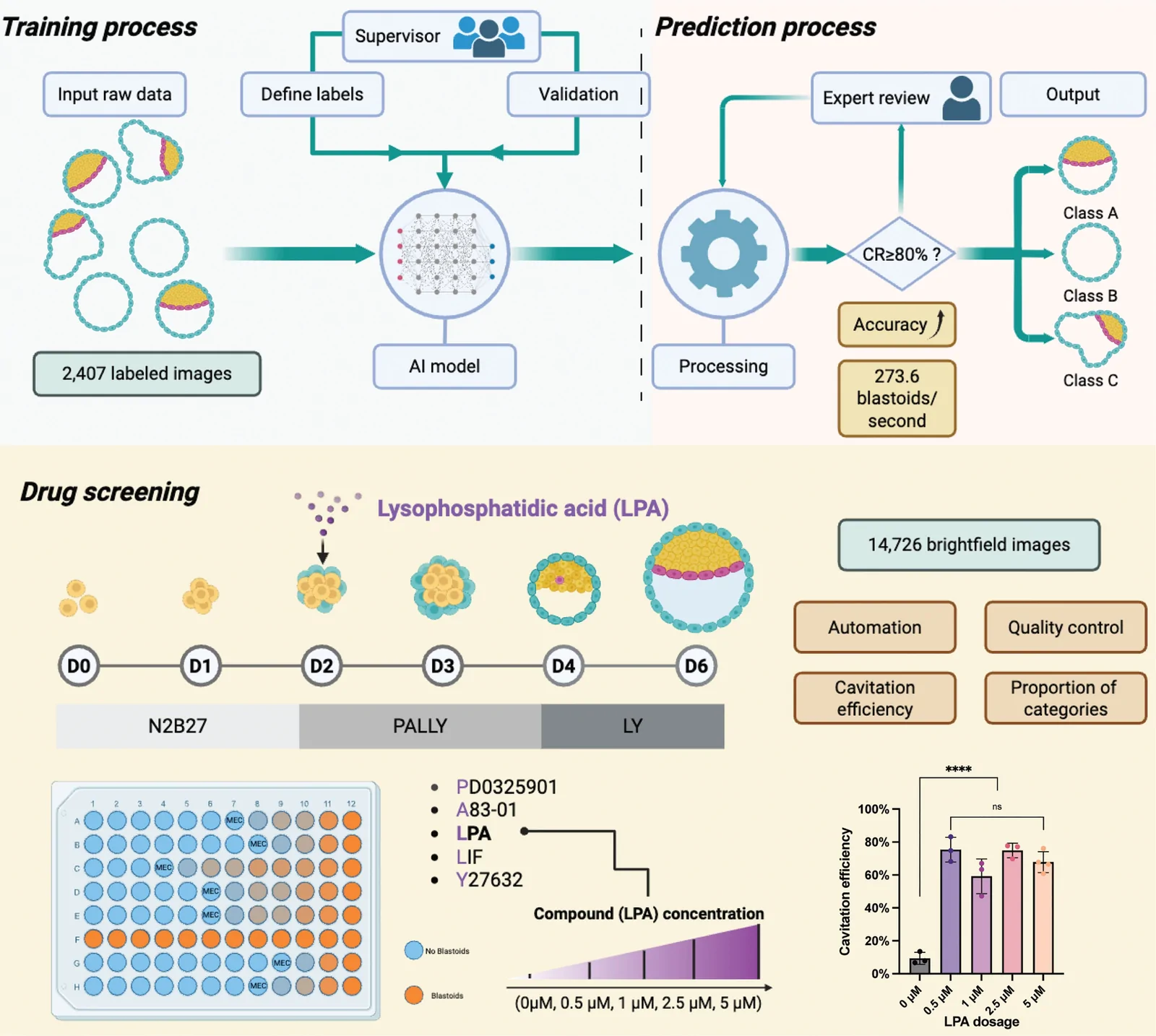
Graphical summary of the deepBlastoid model. The upper panel illustrates the data flow within the deep learning model, while the lower panel depicts its real-world application: a systematic assessment of the effects of lysophosphatidic acid (LPA) concentration on blastoid formation.

To efficiently evaluate morphological features of live blastoids, we developed a deep-learning classifier based on the ResNet-18 architecture (Fig. 6). Using only brightfield images, the model automatically classifies blastoids into five distinct morphological classes. After training, this classifier processes 273.6 blastoid images per second with 87% accuracy. To further minimize misclassification, a CR threshold can be chosen to balance accuracy and throughput, where images with low CR scores are flagged for human expert review. Two use cases were demonstrated using this deep-learning model: (i) the effect of a gradient dosage of LPA on blastoid formation and (ii) the effect of low-dose DMSO on blastoid formation. We found that 0.5 µM LPA significantly promoted Class B blastoid formation. Additionally, 0.1% DMSO had no significant effect on blastoid cavitation compared to the control group. Importantly, the platform also tracked cell seeding density simultaneously, providing a convenient quality control that strengthens the reliability of these experiments.

We generated the first brightfield image dataset of human blastoid models, comprising 17,133 images. Among them, 2407 images were labeled and utilized for model training, validation, and testing. Additionally, 14,726 unlabeled images, capturing various experimental conditions (Fig. 6), were provided as an open resource. All images in the dataset were preprocessed to a standardized resolution of 300 × 300 pixels. This dataset serves as a valuable resource for researchers seeking to optimize model performance or investigate embryo development.

The deepBlastoid model, which is publicly available and adaptable, is designed to be easy to use across research and clinical settings. Researchers can train their own models based on this framework, tailoring it to their specific imaging conditions and blastoid culture protocols. To train a customized model, it is recommended to start with a few hundred images and gradually increase the dataset size as needed until satisfactory accuracy is achieved. Based on our experience, initiating model training with approximately 500 labeled images is a practical starting point for testing feasibility, while using around 1500 images generally results in stable performance. To reduce the risk of overfitting, it is advisable to include data from at least three distinct biological batches. Given that imbalanced datasets are common in real-world blastoid experiments, applying targeted data augmentation to underrepresented classes or increasing dataset diversity can effectively address class imbalance. Furthermore, model evaluation should not rely solely on accuracy; comprehensive metrics such as precision, recall, and F1-score should also be employed to assess the impact of class imbalance and ensure robust performance.

One straightforward application of deepBlastoid is in the assessment of compound toxicity, such as teratogenic effects. In this context, Class B and Class C blastoids represent abnormal morphologies associated with teratogenic outcomes, while Class D reflects cellular debris, which may indicate severe toxicity. Theoretically, exposure to toxic compounds would increase the proportion of Class D. However, this application cannot work without systematic validation and specific characterization of teratogenic effects. Additionally, the proportion of Class W blastoids may serve as a quality control indicator for seeding density, as its ratio remains stable across varying drug concentrations in previous experiments. For mechanism research, the deep-learning model offers a solution by automatically conducting comprehensive evaluations and detailed classifications of blastoids, potentially revealing new phenomena. In drug screening, the deepBlastoid classifier is both labor- and time-efficient, facilitating large-scale assessments of blastoids [26]. It offers a promising approach for evaluating drug effects on embryogenesis and implantation [56].

## Research limitations

Despite its high accuracy and speed, the deepBlastoid model still has some limitations. The dataset used for training, while effective, is relatively small and lacks diversity. While the model achieves 87% accuracy with thousands of images, this accuracy could be improved by incorporating a larger and more varied dataset. Notably, the imbalance in the dataset—particularly the underrepresentation of Class A—has resulted in lower sensitivity for that class, and increasing the proportion of Class A images would likely enhance classification performance. Another limitation is that the biological relevance of the predicted quality categories has not been validated through functional assays; the current reliance on morphology-based labeling may compromise the reliability of the model. Future studies should incorporate functional validation, such as immunostaining or implantation assays, to strengthen biological interpretation. Additionally, to improve the model’s applicability and generalizability, future efforts should involve training with datasets from various imaging conditions [57] cell lines, and blastoid culture protocols. When dealing with a dataset with greater morphological variability or differing imaging conditions, modifications to the model architecture may be necessary, such as the addition of layers to enhance feature extraction. Comprehensive evaluation metrics should also be implemented to ensure robustness across diverse image types. While the current classification into five classes is based on morphology, a detailed classification into more classes could provide a more comprehensive understanding of blastoid development. With respect to the CR ratio, it should be noted that it does not essentially minimize error, it just achieves a balance between accuracy and throughput, thereby making it more accessible for biological applications.

The current study primarily focused on cavitation efficiency, leaving other valuable information unexamined [12, 24]. In the future, beyond cavitation and classification, additional metrics—such as cell number, diameter, and cell type, are obvious targets, which could potentially be inferred from brightfield images in conjunction with synthetic image generation augmentation. If so, the deepBlastoid model would become a powerful, noninvasive tool for high-throughput blastoid evaluation. Moreover, the temporal progression of blastoid formation contains valuable biological insights that could be extracted through the analysis of time-lapse imaging data using deep-learning approaches. The choice of model architecture will depend on what we want from the time-series data: for instance, 3D CNNs [58] and LSTM [59] networks are well-suited for forecasting, while models such as MoCoGAN [60] and TimeSformer [61] are more appropriate for sequence generation and trajectory modeling. The overall framework may be adaptable and holds potential for extension to other 3D stem cell-derived models such as gastruloids or organoids with adequate specific dataset.

## Methods

### Research ethics

This study was reviewed and approved by the KAUST Institutional Biosafety and Bioethics Committee (IBEC), approval number 21IBEC055.

### Study design

This study aimed to develop a deep learning model for accurate and high-throughput classification of blastoids based on brightfield images (Fig. 6). The design, training, and enhancement of the classifier were key components of the study. The deep learning model was constructed using the ResNet-18 architecture. A dataset of over 2000 labeled brightfield images of blastoids, generated through established biological protocols, was used to train the model. The performance of the model was evaluated with key metrics including accuracy, specificity, sensitivity, and loss function. After introducing a minimum CR threshold, the model’s accuracy improved, though throughput decreased slightly. A competition between human experts and the deep learning model further demonstrated the classifier’s superior performance in blastoid classification.

Additionally, the study explored the effect of a gradient dosage of LPA on blastoid formation using the deep learning model. In this field application, blastoids were exposed to gradient LPA concentrations, and the model comprehensively evaluated their formation, with empty ratio serving as a robust quality control measure. In a separate investigation, the model was used to assess the impact of DMSO on blastoid formation by processing image data and evaluating the blastoids. Statistical significance in these analyses was verified using one-way ANOVA.

### Human induced pluripotent stem cells (hiPSCs) and initial culture techniques

The naive pluripotent stem cells (nPSCs) used in this study are chemically reset (cR) cR-SC-9N hiPSCs, which were used in our previous study [24]. The primed PSCs underwent epigenetic resetting following an established protocol with modification [62]. Briefly, primed state PSCs were transitioned onto inactivated mouse embryonic fibroblasts (iMEF) and treated with N2B27 basal medium supplemented with 1 μM PD0325901 (Selleckchem, S1036), 10 ng/mL leukemia inhibitory factor (LIF; Cell Signaling Technology, 62226S), and 1 mM valproic acid (VPA; Sigma-Aldrich, P4543) for 3 days. N2B27 basal medium contains 50% Neurobasal medium (Thermo Fisher Scientific, 21103049) and 50% DMEM/F12 (Thermo Fisher Scientific, 30044333) adding N2 supplement (100×) (Thermo Fisher Scientific, 17502001), B27 without Vitamin A (50X) (Thermo Fisher Scientific, 12587010), 2-mercaptoethanol (Thermo Fisher Scientific, 21-985-023), GlutaMAX (Thermo Fisher Scientific, 35050-010), NEAA (Thermo Fisher Scientific, 11350912), Penicillin/Streptomycin (Thermo Fisher Scientific, 15140-122), and HEPES (Thermo Fisher Scientific, 15630080).

After this initial phase, cells were maintained in N2B27 basal medium supplemented with 1 μM PD0325901, 2 μM XAV-939 (Enzo Life Sciences, BML-WN100-0005), 2 μM Gö6983 (Selleckchem, S2911), and 10 ng/mL LIF (PXGL). By day 10, naïve dome-shaped colonies formed and were subsequently purified through fluorescence-activated cell sorting (FACS) for SUSD2 and stabilized over several passages. The cells were cultured at 37°C under conditions of 7% CO_2_ and 5% O_2_.

### Generation of blastoids

To prepare PXGL-nPSCs for blastoid differentiation, nPSCs were cultured on inactivated mouse embryonic fibroblasts (iMEF) in PXGL medium for 3–4 days. The cells were then dissociated using TrypLE (Life Technologies, 12604-021) for 10 min at 37°C with gentle pipetting. TrypLE was inactivated using 0.2% BSA (HyClone, SH30574.02) in N2B27 basal medium, followed by centrifugation to pellet the cells. To remove iMEF, the cells were seeded onto a 0.1% gelatin-coated plate for 60–90 min. After this, cells were passed through a 40 μm strainer to eliminate clumps, centrifuged again, and resuspended in the appropriate medium for further experiments.

For blastoid formation, we followed an established protocol [15]. Cells were resuspended in N2B27 basal medium supplemented with 10 μM Y-27632 (Selleckchem, S1049) and seeded at a density of approximately 75 cells per microwell in 400 μm AggreWell plates (Stem Cell Technology, cat# 34425). After 2 days, cell aggregates formed. And then medium was changed to N2B27 supplemented with PALLY (1 μM PD0325901, 1 μM A83-01 (Axon Medchem, 1421), LPA (varying concentrations; 5 μM is the condition for immunostaining and post-implantation assay), 10 ng/mL LIF, and 10 μM Y-27632) for two days. Subsequently, the medium was switched to N2B27 supplemented with LPA and 10 μM Y-27632 (LY) for an additional 2 days. As for 0.1% DMSO condition, 0.1%DMSO was added to PALLY and LY medium under 5 μM LPA concentration.

### Brightfield imaging

Blastoid imaging was performed using a Zeiss Cell Discovery 7 (CD7) microscope under brightfield conditions. Due to the dimensions of the AggreWell plate, imaging was conducted at 5× magnification with a 0.5× tube lens. Brightfield mode was employed, and *Z*-focus values were defined for each well, with focus adjustments centered on the well. Although defocusing is difficult to eliminate when capturing large-field tile images, the deep learning model utilized in this study is sufficiently robust to process defocused images. To minimize sample movement, the camera velocity and acceleration were both set to 10%.

### Image cropping

After imaging, the AggreWell plate images, which contain thousands of blastoids, were cropped into smaller images corresponding to the size of individual microwells (Fig. 1B) to facilitate subsequent model training. The cropping was performed using the Cropping tool in Photoshop 2023, allowing for precise and efficient segmentation of the large images. No adjustments were made to contrast, brightness, or rotation. The final processed images had a resolution of approximately 300 × 300 pixels, in 16-bit grayscale, and were saved in PNG format. They are resized to 512 × 512 as the input of the deep learning model. On average, cropping required approximately 10 min per well, with each well yielding around 1200 images—equating to approximately 0.5 s of processing time per image. It is important to note that the time spent on cropping was not included in the downstream classification time. While the current procedure was manually performed, it could be automated using available plugins in Photoshop or other image-processing software.

### Labeling of the dataset

The images were carefully evaluated and labeled by biological experts experienced in embryonic development and blastoid culture. A total of 2407 images from three experimental batches were classified into five typical categories based on developmental features: Class A, B, C, D, and W (Fig. 1B). Class A represents a blastoid with a well-formed cavity and an inner cell mass (ICM) enveloped by a complete circular TE layer. Class B represents a blastoid with a cavity but no ICM. Class C represents a blastoid with an ICM but an irregular or broken TE layer. Class D represents a structure that does not contain a cavity, appearing as cellular debris. Class W represents an empty microwell. Labeling based on immunofluorescence (IF) was excluded, as the IF process can cause morphological and grayscale changes, potentially leading to label mismatches. Image labeling was performed using the Make Sense website.

To ensure labeling accuracy and consistency, three independent experts participated in the annotation process. Inter-rater reliability was assessed among the 2407 labeled images using standard statistical measures, yielding a Fleiss’ Kappa (κ) of approximately 0.92 and a Krippendorff’s Alpha (α) of approximately 0.89, indicating strong agreement and high reliability among raters.

### Cross-entropy loss function

The cross-entropy loss function is widely used in classification tasks, particularly for multi-class classification problems. It measures the difference between the true probability distribution (actual labels) and the predicted probability distribution (model’s output). The goal is to minimize this loss, ensuring the predicted probabilities align closely with the actual labels.

The loss function is *L*:


$$\begin{array}{l} L=-\underset{i=1}{\overset{C}{\sum }}\;y_{i}\log\left(\hat{y_{i}}\right) \end{array}$$

where:

-
$y_{i}$ is the actual label (1 for the correct class, 0 otherwise).
-
$\hat{y_{i}}$ is the predicted probability for class i (output of softmax).
-
C is the number of classes, 5 in our case.

### Model training methods

Several deep learning models, including ResNet variants (18, 50, and 101 layers), EfficientNetV2-S, DenseNet-121, and ViT-Base, were pre-trained on the ImageNet dataset and fine-tuned using our dataset. After evaluating their performance, we selected ResNet18, which demonstrated the best balance between accuracy and throughput. Three batches of blastoid data were used, totaling 2407 images, 300 of which are used as test dataset. 2107 images were randomly divided into a training set with 1796 images and a validation set with 311 images, respectively. To diversify the training data and further improve the model performance and generalization, we adopt various data augmentation strategies during the training stage, including horizontal flip, vertical flip, color jitter, random scaling, and random erasing. The probability for adopting color jitter and random erasing is set to 40% and 20%, respectively. The scaling factor ranges from 0.8 to 1.0. We employ the standard cross-entropy loss and the AdamW optimizer to train our model for 36 epochs with a learning rate of 3e−4 and a weight decay of 1e−3. We clip the gradient to stabilize the training and scale the learning rate of the last fully connected layer with 10 to achieve fast convergence. We selected the model trained with 36 epochs to ensure convergent. The model’s total training time varies from 5–25 min in different architecture (Fig. 2B).

### Model evaluation metric

To assess the model performance, Accuracy, Precision, Recall, and F1-Score (F1) are used. Accuracy is calculated as the number of correct predictions divided by the total number of predictions:


$$\begin{array}{l} Accuracy=\frac{TP+TN}{TP+TN+FP+FN} \end{array}$$

$$\begin{array}{l} Precision=\frac{TP}{TP+FP} \end{array}$$

$$\begin{array}{l} Recall=\frac{TP}{TP+FN} \end{array}$$

with TP = true positives, TN = true negatives, FP = false positives, and FN = false negatives.


$$\begin{array}{l} F1=\frac{2\times Precision\times Recall}{Precision+Recall} \end{array}$$

The F1-score represents the harmonic mean of precision and recall, offering a unified measure that balances both metrics.

### Competition between human experts and AI model

To compare accuracy and throughput, we conducted a competition between human experts and our AI model. Following the blastoid generation protocol, we first constructed a new dataset form a new biological batch. Within a 20-min period, three human experts classified the dataset based on their professional experience. Their accuracy was recorded, and the number of images processed within that time served as their throughput measure.

Similarly, the AI model was employed to classify the same dataset, generating its own accuracy and throughput metrics. Because the model could classify the entire dataset in under 20 min, its throughput in 20 min was estimated using the average inference time for all images in the dataset.

### Confidence rate calculation

For each input image, the model generates probability for all possible classes, which are then combined to compute the CR. The formular of CR are as follows.


$$\begin{array}{l} p_{i}=\frac{P_{i}}{\sum P_{i}},\;\;\;for\;\;\;each\;\;\;i=1,2,3,4,5 \end{array}$$

The $P_{i}$ represents the possibility of five classifications.

And then calculate the mean (μ) and standard deviation (σ):


$$\begin{array}{l} \mu =\frac{1}{5}\sum p_{i} \end{array}$$

$$\begin{array}{l} \sigma =\sqrt{\frac{1}{5}\sum {\left(p_{i}-\mu \right)}^{2}} \end{array}$$

Finally, we will get CR, considering how many standard deviations the highest value is from the mean of the others.


$$\begin{array}{l} Z=\frac{p_{max}-\mu }{\sigma } \end{array}$$

$$\begin{array}{l} CR=\frac{1}{1+e^{-Z}} \end{array}$$

Thus, the confidence rate reflects how certain the model is about its prediction for following analysis.

### Software and hardware

All the model was trained and tested on a NVIDIA A100 80GB GPU. We use Anaconda to organize the experiment environment, including Python 3.10.13, PyTorch 2.1.2, CUDA 12.2, and cuDNN 8.9.2.

### Immunofluorescence staining

Samples were washed with PBS and fixed in 4% paraformaldehyde (PFA) for 30 min. Following fixation, the samples were washed three times with PBS, then permeabilized and blocked using PBS containing 0.4% Triton-X-100 and 6% normal donkey serum for 1 h. Samples were then incubated with the primary antibodies overnight, which were diluted in PBS containing 0.1% Triton-X-100 and 6% normal donkey serum (PBS++). The primary antibodies used included SOX2 (Cell Signaling Technology, MAB4900S, 1:50), OCT4 (R&D Systems, AF1759), GATA3 (R&D Systems, MAB6300), and GATA4 (Cell Signaling Technology, MAB36966S). After primary antibody incubation, samples were washed three times with PBS++ before being incubated with secondary antibodies (1:500) for 1 h. Nuclear staining was performed using DAPI during the final 15 min of secondary antibody incubation, followed by three washes with PBS. Imaging was conducted using a Stellaris 8 confocal microscope, and image processing was carried out using Fiji software.

### *In vitro* culture of human blastoids for post-implantation assay

The *in vitro* implantation assay was performed according to the previously established protocol [12]. Blastoids were manually picked using mouth pipetting under a stereo microscope (Leica M205 FCA). Before use, an 8-well *µ*-slide chamber was pre-coated with fibronectin (Sigma-Aldrich, F0895) diluted at 1:20 in cold DPBS (with Ca^2^^+^ and Mg^2^^+^) and incubated overnight at 4°C. Blastoids were placed into chamber well, which contained IVC1 medium, and incubated at 37°C with 7% CO₂ and 5% O₂. The IVC1 medium consisted of Advanced DMEM/F12 (Thermo Fisher Scientific, 12-634-028) supplemented with the following components: 20% heat-inactivated Fetal Bovine Serum (Thermo Fisher Scientific, 30044333), 2 mM GlutaMAX, 0.5% Penicillin-Streptomycin, 1% ITS-X supplement (Thermo Fisher Scientific, 51500-056), and 1% sodium pyruvate (Thermo Fisher Scientific, 11360070). Additionally, the medium was supplemented with 8 nM β-estradiol (Sigma-Aldrich, E8875), 200 ng/mL Progesterone (Sigma-Aldrich, P0130), 25 μM N-acetyl-L-cysteine (Sigma-Aldrich, A7250) and Y-27632. After 48 h of culture, the IVC1 medium was replaced with the IVC2 medium, which was identical to IVC1, except that 30% Knockout serum replacement (KOSR; Thermo Fisher Scientific, 10828-028) was used instead of 20% FBS. The blastoids were cultured in the IVC2 medium for an additional 96 h.

### Statistical analysis

Statistical analyses in this study were performed with a high degree of rigor and standardization using GraphPad Prism v8.4.2. To determine significant differences between experimental groups, one-way ANOVA was employed, followed by Tukey’s multiple comparisons test for datasets involving three or more groups. For comparisons between two groups, an unpaired, two-tailed Student’s *t*-test was used. The specific statistical tests applied for each analysis are noted in the corresponding figure legends.

All quantitative data are presented as mean values ± standard error of the mean (SEM), unless otherwise stated. To ensure the reliability and reproducibility of results, each experiment was conducted with at least three independent replicates. Micrographs shown are representative of these replicates. Figures include exact *P* values to highlight the statistical significance of the findings. Additionally, to enhance clarity and transparency, details regarding sample sizes and the specific statistical tests employed for each experiment are thoroughly described in the figure legends.

When performing a significance analysis (*P*-value testing) of Competition between human experts and an AI model, at least three data points are generally regarded as the minimum requirement. Accordingly, we ran our model three times to obtain three independent sets of results, ensuring that the statistical analysis would be both robust and meaningful.

## Supplementary Material

lnaf026_suppl_Supplementary_Figures_S1-S3_Table_S1

## Data Availability

The datasets used for training and evaluating the models can be accessed and downloaded from ZENODO and is accessible as of the date of publication. All source code has been deposited in a publicly available repository on GitHub and is accessible as of the date of publication.
