# Supplementary material for: deepBlastoid: a deep learning model for automated and efficient evaluation of human blastoids
Source: Life Med. 2025 Jul 11;4(6):lnaf026. doi: 10.1093/lifemedi/lnaf026 (PMC12732673; doi:10.1093/lifemedi/lnaf026)
Supplement: lnaf026_suppl_Supplementary_Figures_S1-S3_Table_S1 [file lnaf026_suppl_supplementary_figures_s1-s3_table_s1.docx]

# Supplementary Files

deepBlastoid: A Deep Learning Model for Automated and Efficient Evaluation of Human Blastoids

Zejun Fan^1,3,#^, Zhenyu Li^2,#^, Yiqing Jin^3,#^, Arun Pandian Chandrasekaran^3^, Ismail M. Shakir^3^, Yingzi Zhang^3^, Aisha Siddique^3^, Mengge Wang^3^, Xuan Zhou^3^, Yeteng Tian^3^, Peter Wonka ^2,*^, Mo Li^1, 3, 4,*^

^1^Bioengineering Program, Biological and Environmental Science and Engineering Division (BESE), King Abdullah University of Science and Technology (KAUST), Thuwal 23955, Saudi Arabia

^2^Computer Science Program, Computer, Electrical and Mathematical Science and Engineering Division (CEMSE), King Abdullah University of Science and Technology (KAUST), Thuwal 23955, Saudi Arabia

^3^Bioscience Program, Biological and Environmental Science and Engineering Division (BESE), King Abdullah University of Science and Technology (KAUST), Thuwal 23955, Saudi Arabia

^4^KAUST Center of Excellence for Smart Health (KCSH), Thuwal 23955, Saudi Arabia

^#^These authors contributed equally to this work.

^*^Correspondence: mo.li@kaust.edu.sa (M.L.), [peter.wonka@kaust.edu.sa](mailto:peter.wonka@kaust.edu.sa) (P.W.)


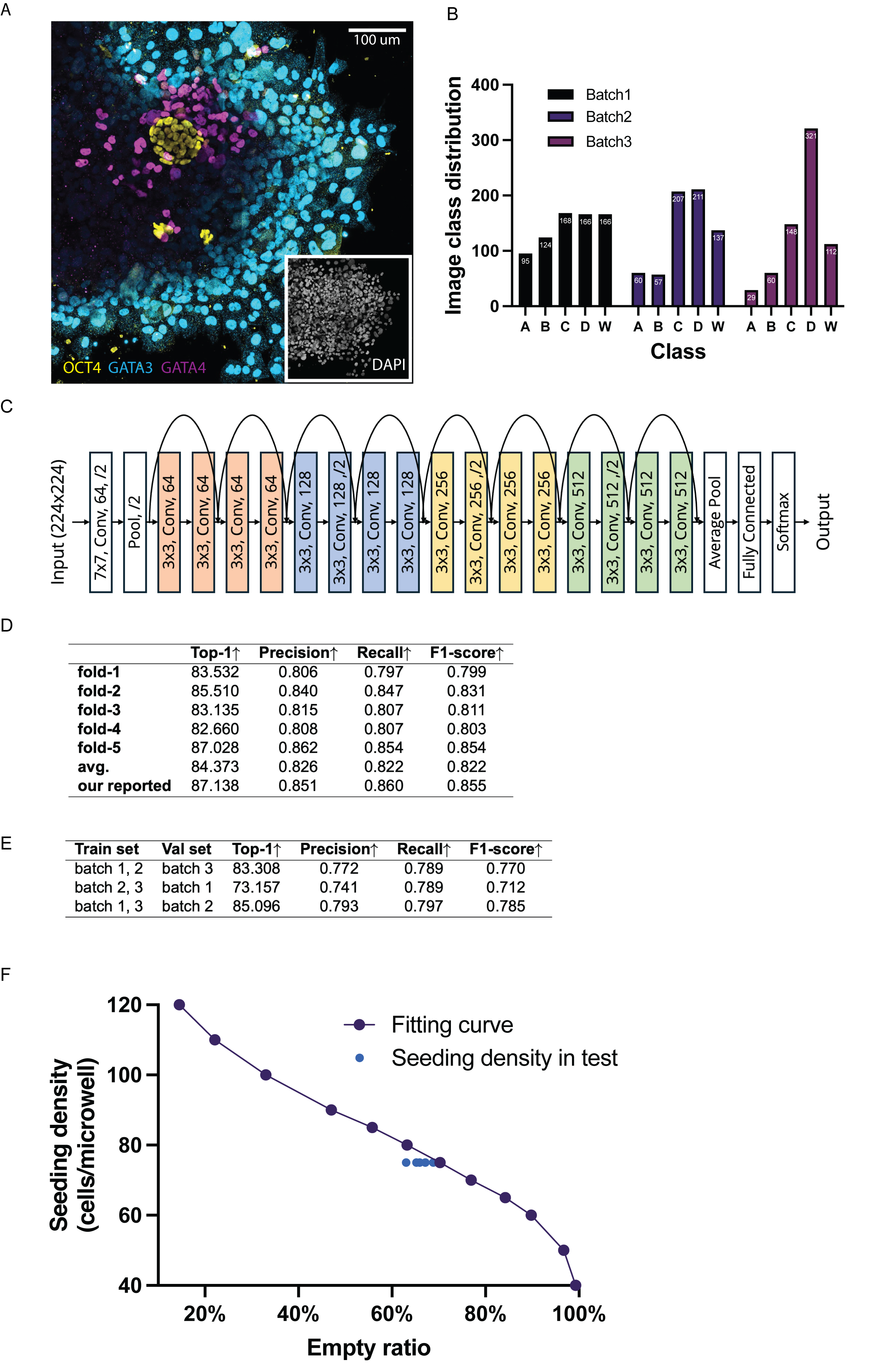


**Supplementary Figure 1.** (A) Immunofluorescence analysis of post-implantation structures reveals the expression of OCT4 (yellow), GATA3 (cyan), and GATA4 (magenta). Scale bar, 100 μm. (B) Distribution histogram of blastoids images in three individual batches. The number of each category is annotated above. (C) Layer information of ResNet-18 convolutional neural network. (D) Table of ResiNet18 performance of with 5-fold experiment. (E) ResNet-18 evaluation results in a round robin fashion. For each time, we select data from two batches as the training time and the left one as the validation set. The second line indicates a potential batch bias in this case. (F) Relationship of empty ratio and seeding density. The purple curve is the calculated curve of seeding density curve and empty ratio. The blue point is for the data range of LPA test, where cell seeding density is 75 cells/microwell. The predicted cell seeding density is 72.9 cells/microwell.


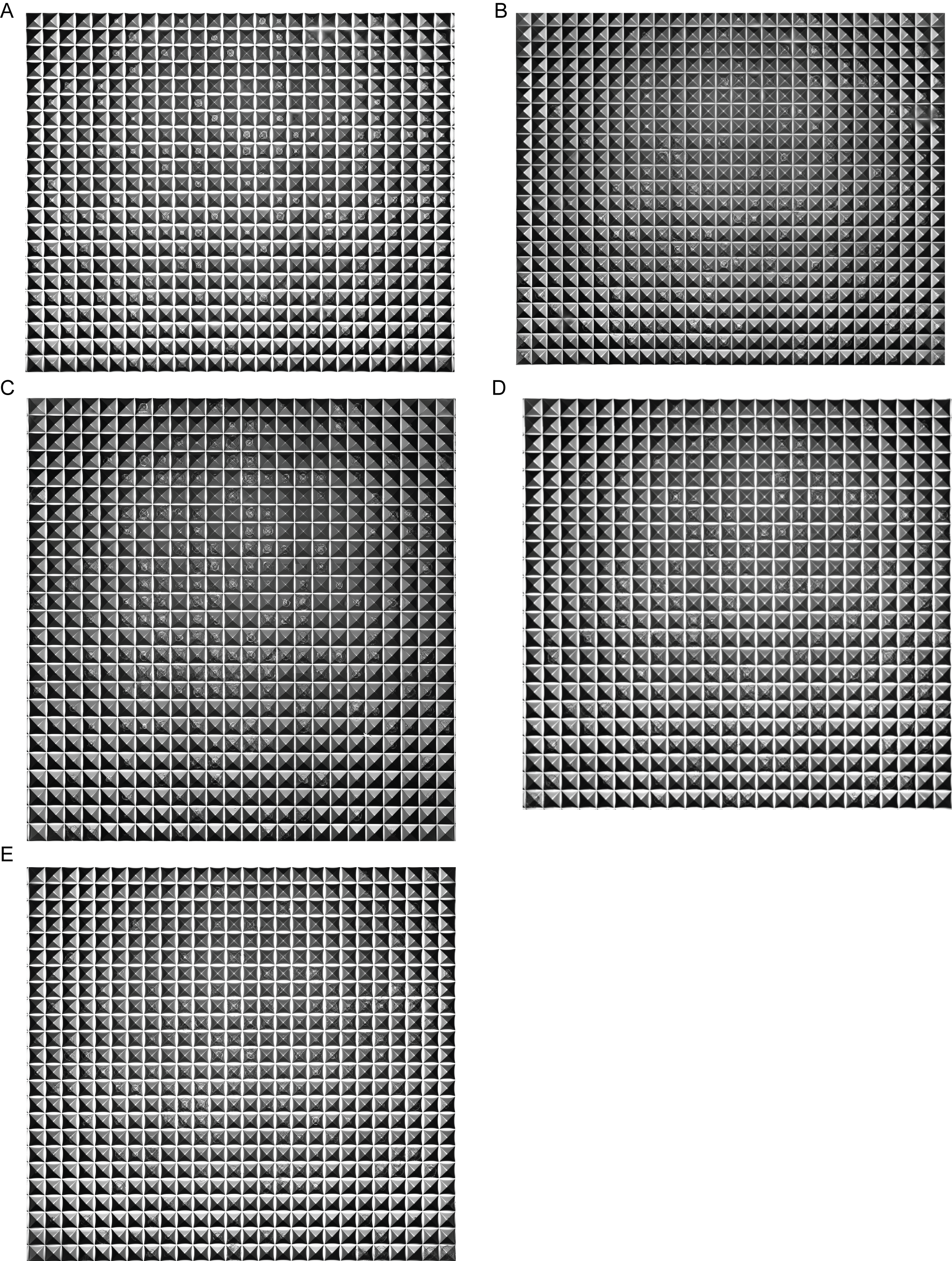


**Supplementary Figure 2. Morphology of blastoid in Aggrewell on dose-effect of LPA.**

(A) 0 μM LPA. (B) 0.5 μM LPA. (C) 1 μM LPA. (D) 2.5 μM LPA. (E) 5 μM LPA.


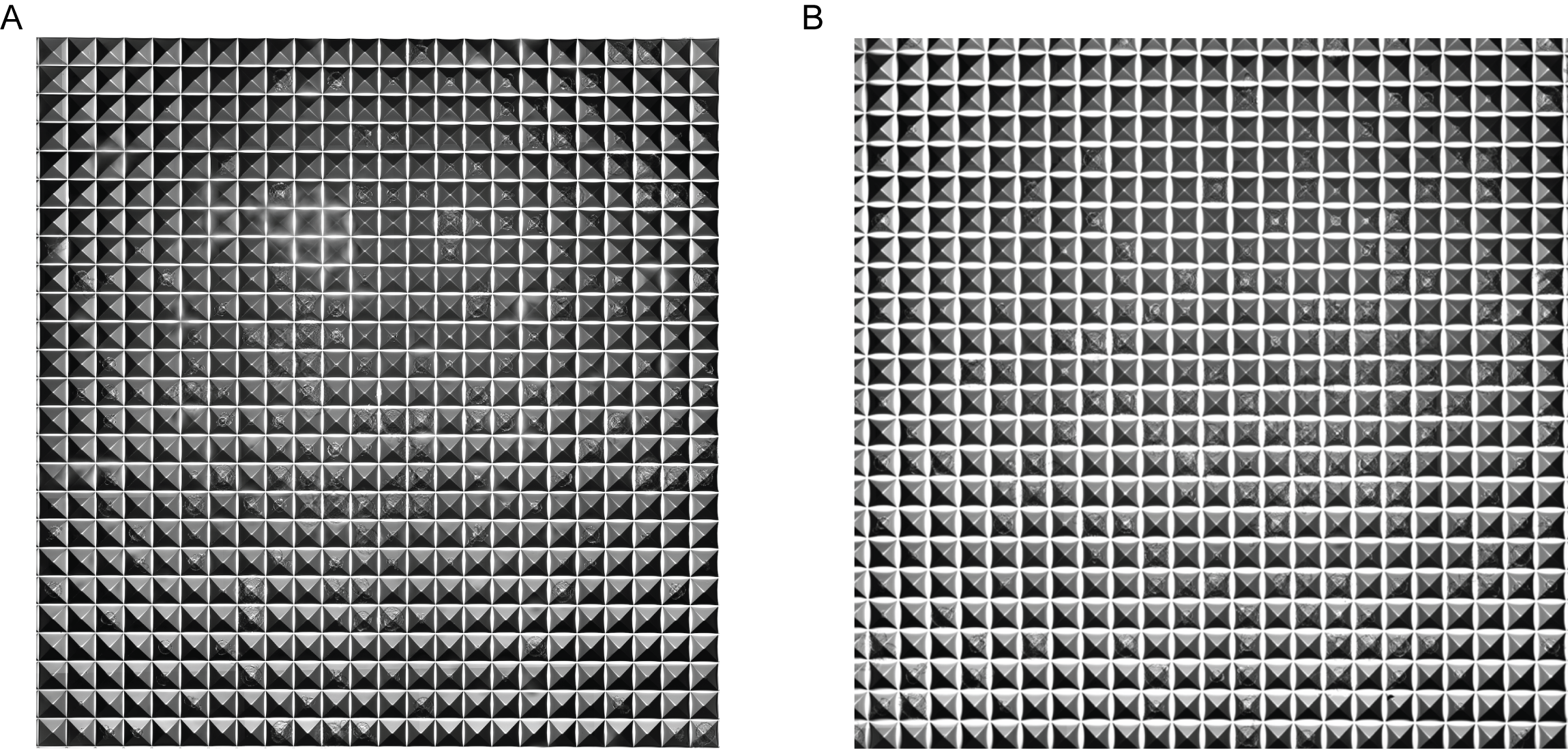


**Supplementary Figure 3. Morphology of blastoid in Aggrewell on the effect of 0.1%DMSO.**

(A) Control group. (B) 0.1%DMSO group.

**Supplementary Table 1**. Key layer information of ResNet-18 convolutional neural network for a 300 × 300 grayscale image input.

| **Layer** | **Width** | **Height** | **Depth** | **Filter height** | **Filter width** |
| --- | --- | --- | --- | --- | --- |
| Input | 521 | 521 | 1 | - | - |
| Conv1 | 256 | 256 | 64 | 7 | 7 |
| Max Pool | 128 | 128 | 64 | 3 | 3 |
| Layer1 (1st ResBlock) | 128 | 128 | 64 | 3 | 3 |
| Layer1 (2nd ResBlock) | 128 | 128 | 64 | 3 | 3 |
| Layer2 (1st ResBlock) | 64 | 64 | 128 | 3 | 3 |
| Layer2 (2nd ResBlock) | 64 | 64 | 128 | 3 | 3 |
| Layer3 (1st ResBlock) | 32 | 32 | 256 | 3 | 3 |
| Layer3 (2nd ResBlock) | 32 | 32 | 256 | 3 | 3 |
| Layer4 (1st ResBlock) | 16 | 16 | 512 | 3 | 3 |
| Layer4 (2nd ResBlock) | 16 | 16 | 512 | 3 | 3 |
| Global Avg Pool | 1 | 1 | 512 | - | - |
| Fully Connected | - | - | 5 | - | - |
